# Tirzepatide for treatment of postbariatric hypoglycemia after Roux-en-Y gastric bypass: a report of 3 cases

**DOI:** 10.1210/jcemcr/luag216

**Published:** 2026-09-03

**Authors:** Jovan Milosavljevic, Caroline Meyer, Joshua A Levine, Saachi Sachdev

**Affiliations:** Division of Endocrinology, Department of Medicine, Montefiore Medical Center, Bronx, NY 10467, USA; Department of Medicine, Albert Einstein College of Medicine, Bronx, NY 10461, USA; Department of Medicine, Albert Einstein College of Medicine, Bronx, NY 10461, USA; Division of Endocrinology, Department of Medicine, Montefiore Medical Center, Bronx, NY 10467, USA; Division of Endocrinology, Department of Medicine, Montefiore Medical Center, Bronx, NY 10467, USA; Department of Medicine, Albert Einstein College of Medicine, Bronx, NY 10461, USA

**Keywords:** tirzepatide, postbariatric hypoglycemia, gastric bypass, GLP-1/GIP, incretins, bariatric surgery

## Abstract

Postbariatric hypoglycemia (PBH) represents a challenging complication of bariatric surgery. This case series describes 3 patients with PBH following Roux-en-Y gastric bypass who were treated with tirzepatide, a glucagon-like peptide-1 (GLP-1) and glucose-dependent insulinotropic polypeptide (GIP) receptor agonist (RA). All patients presented with symptomatic hypoglycemia characterized by episodes of diaphoresis, visual disturbances, confusion, and documented low blood glucose levels occurring postprandially. Conventional management strategies, including dietary modifications, acarbose, and other pharmacotherapies, were either partially effective or limited by side effects. Initiation of tirzepatide led to increased time in range, reduced number and severity of hypoglycemic episodes, and decreased glucose variability. This report suggests that GLP-1 and GIP RA can be a promising adjunct in managing PBH; however, further studies to confirm their efficacy and establish optimal dosing strategies are needed.

## Introduction

Roux-en-Y gastric bypass (RYGB) remains a staple in obesity management, accounting for approximately 25% of all bariatric surgeries performed worldwide [1]. A recognized complication of RYGB is postbariatric hypoglycemia (PBH), which occurs 6 to 12 months postoperatively [2], with prevalence ranging from 0.1% to 0.2% in large national cohort studies [3, 4] to as high as 30% in self-reported surveys [5].

The pathophysiology of PBH is multifactorial and incompletely understood. In adults without type 2 diabetes, nutrient ingestion leads to the release of the incretin hormones glucagon-like peptide-1 (GLP-1) and glucose-dependent insulinotropic polypeptide (GIP). These 2 hormones lead to an enhancement of glucose-dependent insulin secretion, accounting for approximately two-thirds of postprandial insulin release [6]. Additionally, GIP increases glucagon secretion during times of hypoglycemia, while GLP-1 suppresses glucagon during hyperglycemic states [6]. Following RYGB, structural changes to the gut increase the rate of gastric emptying, resulting in a 10-fold increase in GLP-1 release [7]. This acute rise in GLP-1 induces a disproportionately large insulin secretion that becomes decoupled from the gradual postprandial rise in blood glucose, thereby precipitating hypoglycemia 1 to 3 hours after meals [8, 9]. Furthermore, reduced postprandial GIP levels following RYGB may contribute to PBH by diminishing the glucagon secretion normally stimulated by GIP during hypoglycemic episodes [10].

Current treatment strategies for PBH have largely centered on dietary modifications, pharmacotherapy, and surgical interventions, yet complete elimination of hypoglycemia remains challenging with these approaches [11]. Pharmacologic options include medications that delay and reduce glucose absorption, such as acarbose, as well as medications that suppress insulin secretion, including diazoxide, somatostatin analogs, and calcium channel blockers [11, 12]. Although these medications improve PBH to varying degrees, their use is often limited by undesirable side effects that can potentially impact patients’ daily activities and prevent medication adherence [12].

More recently, GLP-1 receptor agonists (GLP-1 RAs) have emerged as a novel PBH treatment strategy [13]. GLP-1 agonists slow gastric emptying, which attenuates the postprandial insulin surge, thereby preventing hypoglycemia. They also stabilize blood glucose, limiting hyperglycemic peaks and preventing rebound hypoglycemia. However, clinical studies have yielded mixed outcomes [14]. Building on the pathophysiology behind PBH, dual incretin RA targeting both GLP-1 and GIP actions may be a promising approach for managing this challenging condition. While there is data showing success of tirzepatide in treating early dumping syndrome and PBH after sleeve gastrectomy [15], to our knowledge, there are no published reports of its use for PBH following RYGB. Here, we report 3 cases of hypoglycemia post RYGB treated with tirzepatide, a GLP-1 and GIP RA.

## Case presentation

Case 1: A 44-year-old woman with a history of asthma, migraine, and obesity status post sleeve gastrectomy converted to RYGB for hiatal hernia presented with postprandial episodes of sweating, visual changes (“everything turning black”), dizziness, and documented hypoglycemia and glucose of 54 mg/dL (SI: 3.0 mmol/L) (reference range, 70-99 mg/dL [SI: 3.9-5.5 mmol/L]), relieved by carbohydrate ingestion. These symptoms began a few months after her bypass surgery, leading to a diagnosis of PBH based upon continuous glucose monitor (CGM) readings, for which dietary modifications were recommended. Previous interventions with acarbose (titrated to 50 mg with meals) and liraglutide (titrated to 1.8 mg daily) were discontinued due to gastrointestinal intolerance, the burden of daily injections, and incomplete relief of symptoms, while diazoxide and calcium channel blockers were deferred because of her baseline hypotension and orthostatic symptoms. Initiation of tirzepatide at 2.5 mg initially improved her hypoglycemia, although intermittent symptoms persisted despite continued dietary adherence.

Case 2: A 47-year-old woman with a history of type 2 diabetes, depression, and obesity status post RYGB developed recurrent episodes of postprandial diaphoresis, confusion, palpitations, and documented severe hypoglycemia as low as 32 mg/dL (SI: 1.8 mmol/L), approximately 6 months after surgery. Following bariatric surgery, her hemoglobin A1c (HbA1c) remained under 5.7% (SI: <39 mmol/mol) (reference range, <5.7% [SI: <39 mmol/mol]) off any glucose-lowering medications. Over time, her symptoms intensified, ultimately occurring on a daily basis and severely impairing her quality of life (QoL) and work capacity. She was unable to work or drive because of the fear of hypoglycemia, ultimately leading to her approval for disability benefits. A mixed meal test confirmed postprandial hypoglycemia. Previous management included dietary modifications, acarbose (25 mg with meals), and semaglutide (titrated to 0.5 mg weekly) with limited success. Transition from semaglutide to tirzepatide 5 mg, which was then increased to 7.5 mg weekly, markedly reduced hypoglycemic events. Although fear of hypoglycemia persisted, she was able to travel for the first time in years.

Case 3: A 54-year-old woman with a history of type 2 diabetes mellitus, obesity status post sleeve gastrectomy, converted to RYGB 3 years later, hypertension, osteopenia, stage 3 chronic kidney disease, and seizure disorder developed symptomatic hypoglycemia approximately 1 year after RYGB. Oral glucose tolerance testing revealed a 2-hour blood glucose of 57 mg/dL (SI: 3.2 mmol/L), despite being asymptomatic at that time. Initial management with dietary modifications and acarbose (titrated to 50 mg with meals) resulted in only partial benefit and was limited by gastrointestinal side effects. Given the persistence of hypoglycemic episodes, tirzepatide 2.5 mg was initiated, which led to the resolution of postprandial hypoglycemia.

## Diagnostic assessment

All 3 cases had a history and presentation clinically consistent with PBH. Case 2 underwent mixed-meal tolerance testing, resulting in insulin of 39 μU/mL (SI: 271 pmol/L) (reference, <3 μU/mL [SI: <21 pmol/L]), C-peptide of 9.4 ng/mL (SI: 3.11 nmol/L) (reference, <0.6 ng/mL [SI: <0.20 nmol/L]), and proinsulin of 28 pmol/L (reference <5 pmol/L) at glucose nadir (38 mg/dL [SI: 2.1 mmol/L]).

Case 3 had documented reactive hypoglycemia (oral glucose tolerance test [OGTT] glucose 57 mg/dL [SI: 3.2 mmol/L]). Case 1 was diagnosed based on suggestive postprandial hypoglycemia patterns on CGM readings. Table 1 shows relevant initial diagnostic laboratory data. Incidentally identified bilateral low attenuation nonfunctioning adrenal adenomas and a gallium-68-DOTATATE positron emission tomography/computed tomography non-avid pancreatic cyst in Case 2 were considered unrelated.

**Table 1:** Initial laboratory data

Laboratory result	Case 1	Case 2	Case 3	Reference range
Hemoglobin A1c	4.7% (28 mmol/mol)	5.0% (31 mmol/mol)	5.1% (32 mmol/mol)	<5.7% (<39 mmol/mol)
TSH	1.87 mIU/L	0.7 mIU/L	0.47 mIU/L	0.30-4.20 mIU/L
8 Am cortisol	9.3 μg/dL (257 nmol/L)	11.5 μg/dL (317 nmol/L)	—	4.0-20.0 μg/dL (110-552 nmol/L)
ACTH	10 pg/mL (2.2 pmol/L)	—	—	7.2-63.3 pg/mL (1.6-14 pmol/L)
DHEA-S	68 μg/dL (185 nmol/L)	153 μg/dL (415 nmol/L)	158 μg/dL (429 nmol/L)	35-430 μg/dL (95-1170 nmol/L)
LDL	86 mg/dL (2.22 mmol/L)	79 mg/dL (2.04 mmol/L)	76 mg/dL (1.97 mmol/L)	<130 mg/dL (<3.37 mmol/L)
Triglycerides	50 mg/dL (0.56 mmol/L)	75 mg/dL (0.85 mmol/L)	**215 **mg/dL **(2.43 **mmol/L**)**	<150 mg/dL (<1.69 mmol/L)
eGFR	92 mL/min/1.73 m^2^	91 mL/min/1.73 m^2^	**36 **mL/min/1.73 m^2^	>60 mL/min/1.73 m^2^
AST	28 U/L	21 U/L	31 U/L	<37 U/L
ALT	26 U/L	<10 U/L	18 U/L	10-49 U/L
Total bilirubin	0.6 mg/dL	0.3 mg/dL	0.3 mg/dL	0.3-1.2 mg/dL

Abbreviations: ACTH, adrenocorticotropic hormone; ALT, alanine transaminase; AST, aspartate aminotransferase; DHEA-S, dehydroepiandrosterone sulfate; eGFR, estimated glomerular filtration rate; LDL, low-density lipoprotein; TSH, thyrotropin (thyroid-stimulating hormone).

Abnormal values are shown in bold font. Values in parentheses are International System of Units (SI).

## Treatment

Case 1: Tirzepatide was initiated at 2.5 mg weekly and increased to 5 mg weekly at month 4. At month 11, the dose was reduced back to 2.5 mg weekly due to excessive weight loss.

Case 2: Tirzepatide was initiated at 5 mg weekly and later increased to 7.5 mg weekly at month 1. She continued to take acarbose 25 mg with meals.

Case 3: Tirzepatide was initiated at 2.5 mg weekly and increased to 5 mg weekly at month 3. Acarbose 50 mg with meals was discontinued due to symptom resolution.

All 3 patients received individualized dietary counseling per postbariatric hypoglycemia principles: approximately 30 g of low-glycemic-index complex carbohydrate per meal and 15 g per snack, each paired with protein and fat, avoiding simple/high-glycemic-index carbohydrates and caloric beverages [16].

## Outcome and follow-up

**Figure 1:**
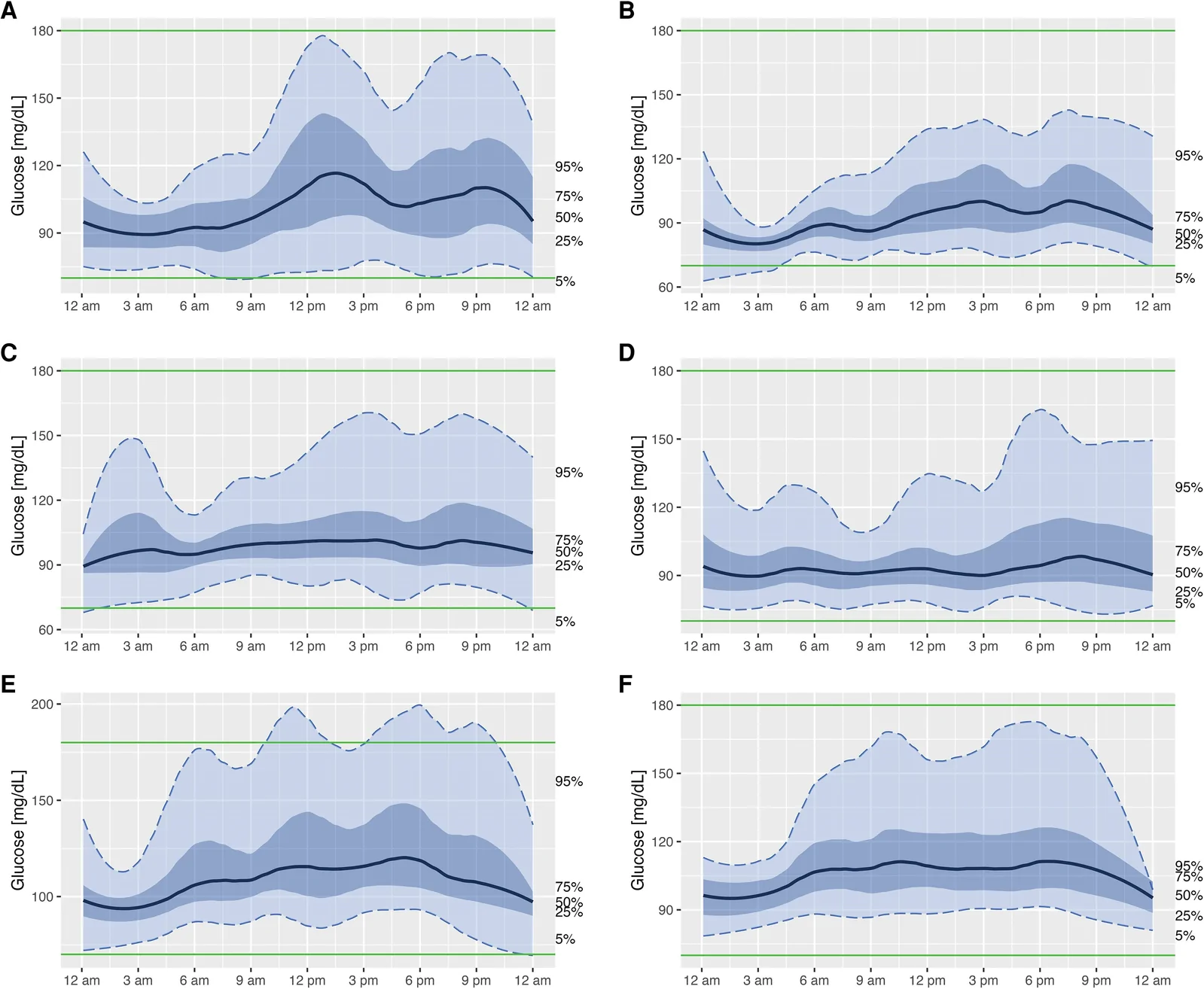
Thirty-day ambulatory glucose profile before and after initiation of tirzepatide for all 3 cases. Panels show Case 1 (A, pre; B, post), Case 2 (C, pre; D, post), and Case 3 (E, pre; F, post). The thick solid line is the median glucose at each time of day; the dark blue band is the interquartile range (25th-75th percentile); the light blue band spans the 5th to 95th percentiles (with dashed lines marking the 5th and 95th). Green horizontal lines denote the hypoglycemia (70 mg/dL) and hyperglycemia (180 mg/dL) thresholds. Data are aggregated across all CGM readings over the study period. Profiles were produced in R using the iglu package [24].

Figure 1 shows 30-day CGM ambulatory glucose profiles, and Table 2 summarizes CGM metrics. In Case 1, intermittent symptomatic hypoglycemia persisted despite tirzepatide and strict dietary adherence. Additionally, she experienced weight loss from 180 to 147 lbs, with BMI decreasing from 31.0 kg/m^2^ at tirzepatide initiation to 25.1 kg/m^2^ at 6-month follow-up. Case 2 demonstrated significant clinical improvement with tirzepatide, with a reduced frequency and severity of hypoglycemic episodes. BMI decreased from 33.5 kg/m^2^ at tirzepatide initiation to 27.5 kg/m^2^ at 6-month follow-up. In Case 3, tirzepatide led to the complete resolution of symptomatic hypoglycemia, a 20-lb weight loss, a BMI decrease from 33.0 to 26.0 kg/m^2^, and triglyceride improvement from 215 to 93 mg/dL (SI: 2.43 to 1.05 mmol/L) at 6 months. Acarbose was discontinued. Importantly, all patients reported marked improvement in QoL and restoration of normal daily functioning following initiation of tirzepatide. No significant adverse events directly attributed to tirzepatide were observed in any case.

**Table 2:** Continuous glucose monitor (CGM) metrics before and after starting tirzepatide

CGM metrics	Case 1		Case 2		Case 3	
	Pre	Post	Pre	Post	Pre	Post
**Number of days**	31 days	31 days	31 days	31 days	31 days	31 days
**CGM active time**	96.4%	87.8%	96.1%	91.4%	84.8%	99.5%
**GMI**	5.8%	5.6%	5.8%	5.6%	6.1%	5.9%
**CV**	26.3%	20.7%	22.2%	22.5%	28.9%	21.5%
**SD**	27.6 mg/dL	19.5 mg/dL	22.8 mg/dL	21.9 mg/dL	33.4 mg/dL	23.6 mg/dL
**MAGE**	71.5 mg/dL	53.4 mg/dL	64.6 mg/dL	61.9 mg/dL	95.6 mg/dL	69.3 mg/dL
**Time below 54 mg/dL**						
Overall	0.7%	0.4%	0.5%	0.1%	0.3%	0%
Daytime only	1.0%	0.1%	0.4%	0.1%	0.3%	0%
**Time below 70 mg/dL**						
Overall	4.7%	3.4%	2.5%	1.5%	1.9%	0.2%
Daytime only	5.0%	2.0%	1.8%	1.5%	2.0%	0.1%
**Time in range (70-180 mg/dL)**	93.3%	96.5%	96.2%	97.3%	92.2%	97.5%
**Time above 180 mg/dL**	2.0%	0.1%	1.3%	1.1%	5.9%	2.3%
**Time above 250 mg/dL**	0.0%	0.0%	0.0%	0.1%	0.5%	0.0%
**Level 1 hypoglycemia events (<70 mg/dL)**						
Number of episodes	55	35	25	16	20	4
Mean duration of episode	35.4 min	34.1 min	35.4 min	24.4 min	30.2 min	20.0 min
Mean glucose during the episode	62.3 mg/dL	62.6 mg/dL	60.7 mg/dL	64.0 mg/dL	61.5 mg/dL	65.2 mg/dL
Number of episodes - daytime only	44	16	14	10	13	1
Mean duration of episode - daytime only	31.1 min	25.0 min	25.4 min	25.5 min	36.5 min	15.0 min
Mean glucose during the episode - daytime	62.4 mg/dL	63.6 mg/dL	59.8 mg/dL	62.4 mg/dL	62.2 mg/dL	68.4 mg/dL
**Level 2 hypoglycemia events (<54 mg/dL)**						
Number of episodes	9	5	6	0	3	0
Mean duration of episode	28.3 min	22.0 min	37.5 min	0.0 min	26.7 min	0.0 min
Mean glucose during the episode	45.1 mg/dL	49.4 mg/dL	43.2 mg/dL	NA	49.9 mg/dL	NA
Number of episodes - daytime only	7	2	3	0	2	0
Mean duration of episode - daytime only	32.1 min	15.0 min	33.3 min	0.0 min	30.0 min	0.0 min
Mean glucose during the episode - daytime (mg/dL)	45.3 mg/dL	50.9 mg/dL	42.4 mg/dL	NA	53.5 mg/dL	NA

Abbreviations: GMI, glucose management indicator; CV, coefficient of variation; SD, standard deviation; MAGE, mean amplitude of glycemic excursions; NA, not applicable.

To convert glucose from mg/dL to SI units (mmol/L), multiply by 0.0555.

For all three patients, CGM data were obtained from two time periods: the month prior to tirzepatide initiation and the month preceding the 6-month follow-up visit. Post-treatment CGM was obtained on tirzepatide 5 mg weekly (Case 1), 7.5 mg weekly (Case 2), and 5 mg weekly (Case 3). Acarbose (25 mg with meals) was used concurrently during the post-treatment CGM period in Case 2 only; Case 1 received no acarbose, and in Case 3 acarbose had been discontinued.

Glucose metrics were calculated using the R package iglu (24). Daytime metrics were defined as those recorded between 8:00 AM and midnight, broadly representing the hours during which meal-induced hypoglycemia may occur (25). Hypoglycemic episode was defined as any continuous period lasting at least 15 minutes.

## Discussion

These 3 cases illustrate successful use of tirzepatide in the management of PBH. Following treatment, patients experienced increased time in range and reduced hyperglycemic excursions. Additionally, both the number and duration of hypoglycemic episodes, especially level 2 events (<54 mg/dL [SI: <3.0 mmol/L]), decreased substantially, with daytime episodes nearly eliminated in some cases. Overall, tirzepatide reduced glycemic variability, as evidenced by lower coefficients of variation, standard deviations, and mean amplitude of glycemic excursions [17]. To our knowledge, this report is the first to document tirzepatide use for PBH after RYGB. Notably, Stortz and Lawler [15] previously showed its success in treating early dumping syndrome and PBH after sleeve gastrectomy.

The role of incretins in PBH remains incompletely understood. In mixed-meal tolerance testing (MMTT), patients who underwent RYGB exhibit an exaggerated acute GLP-1 rise, which further increases insulin secretory response [18]. Accelerated nutrient delivery to the small intestine leads to rapid absorption and subsequent rapid rise in postprandial plasma glucose levels, further stimulating insulin secretion [19]. Lastly, postbariatric surgery patients experience reduced counterregulatory responses, including lower levels of glucagon, cortisol, and catecholamines, with diminished autonomic outflow during hypoglycemia [20]. The role of GLP-1 RA is not fully defined either. It has been suggested that continuous stimulation of GLP-1 receptors by exogenous GLP-1 may benefit postprandial hypoglycemia [9]. Additionally, these agents slow gastric emptying [21], which may reduce postprandial glucose excursions and, in turn, reduce the chance of reactive hypoglycemia, although this effect diminishes over time [6]. Furthermore, prior studies have shown that in hypoglycemic states, GLP-1 RA may increase glucagon response and suppress insulin secretion [22]. Nevertheless, case reports and small studies have not consistently demonstrated a benefit of GLP-1 agonists for PBH. In one open, uncontrolled observational study, 5 PBH patients were successfully treated with liraglutide [13]. In contrast, a small randomized crossover study of 11 participants showed no reduction in hypoglycemia with liraglutide after MMTT [12]. However, CGM data revealed lower variability indices and reduced time with hyperglycemia. Therefore, the authors suggested that liraglutide may act as a blood glucose stabilizer [12].

The role of GIP in PBH remains understudied. In normo- and hyperglycemic states, GIP does not affect glucagon secretion. However, during hypoglycemia, GIP increases glucagon levels and may serve as a glucose stabilizer [23]. Previous research has shown that patients after RYGB tend to have low GIP levels [10]. Moreover, reversing RYGB increased plasma GIP levels and resolved hypoglycemia in a patient with severe PBH [19]. These findings suggest that low GIP levels may play a role in this condition, supporting the potential role of tirzepatide as a therapeutic agent.

These findings suggest that tirzepatide may effectively reduce PBH both by reducing hyperglycemia, which in turn prevents the cascade of reactive hypoglycemia, and increasing glucagon levels during hypoglycemia, thereby resulting in overall stabilization of glucose levels. Future prospective studies should confirm its efficacy, define optimal dosing, and assess comprehensive patient outcomes, including QoL metrics, to establish its long-term benefit.

## Learning points

- The role of incretins in PBH remains incompletely understood. Post RYGB patients exhibit an exaggerated rapid GLP-1 response following MMTT, which may stimulate increased insulin secretion and lead to hypoglycemia.
- The role of GIP in PBH is under-researched; however, reduced postprandial GIP levels post RYGB may contribute to PBH by diminishing glucagon secretion normally stimulated by GIP during hypoglycemic episodes.
- Conventional treatments for PBH can be limited by incomplete efficacy and side effects.
- Continuous glucose monitoring is essential for assessing treatment response, frequency, and severity of hypoglycemic events.
- Tirzepatide may reduce both the frequency and severity of PBH episodes, and the dual action on GLP-1 and GIP receptors offers a novel mechanism to manage reactive hypoglycemia.

## Data Availability

The de-identified CGM data are available from the corresponding author upon reasonable request.
